# Concurrent Tuberculous Meningitis and Toxoplasma Encephalitis in an HIV-Positive Patient: An Exceptionally Rare Case

**DOI:** 10.7759/cureus.91578

**Published:** 2025-09-04

**Authors:** Lizi Adishvili, Shorena Dvali

**Affiliations:** 1 Medicine, David Tvildiani Medical University, Tbilisi, GEO; 2 HIV/AIDS Department, Infectious Diseases, AIDS and Clinical Immunology Research Center, Tbilisi, GEO

**Keywords:** advanced hiv disease, central nervous system tuberculosis, cns toxoplasmosis, hiv aids, tuberculous meningitis (tbm)

## Abstract

Human immunodeficiency virus (HIV) infection is strongly associated with an increased risk of opportunistic infections affecting the central nervous system (CNS). Among these, tuberculous meningitis (TBM) and Toxoplasma encephalitis (TE) represent two of the most frequent and severe infections seen in individuals with advanced immunosuppression. Each condition alone is associated with significant morbidity and mortality, particularly in patients with profoundly diminished CD4+ T-cell counts. While TBM and TE are common individually in the context of HIV/AIDS, their concurrent occurrence within the CNS appears to be exceptionally rare, with only rare or isolated reports in the literature. The co-existence of these infections poses substantial diagnostic and therapeutic challenges due to overlapping clinical manifestations, radiological findings, and the potential for rapid neurological decline.

We report a case of a 46-year-old woman with long-standing HIV/AIDS, non-adherent to antiretroviral therapy, who presented with fever, headache, confusion, vomiting, and progressive neurological deficits. Cerebrospinal fluid analysis revealed lymphocytic pleocytosis with low glucose levels and a positive GeneXpert test for Mycobacterium tuberculosis. Toxoplasma IgG was markedly elevated, and brain MRI demonstrated multiple ring-enhancing lesions with surrounding vasogenic edema. Laboratory investigations confirmed advanced immunosuppression, with a declining CD4+ T-cell count and high viral load. She was treated with a combination of anti-tuberculous drugs, corticosteroids, and anti-Toxoplasma therapy. Despite treatment, her neurological condition deteriorated, with residual motor and cognitive deficits at discharge.

## Introduction

Human immunodeficiency virus (HIV) infection has long been associated with an increased susceptibility to a wide array of opportunistic infections, particularly those affecting the central nervous system (CNS). Among these, tuberculous meningitis (TBM) and Toxoplasma encephalitis (TE) are among the most frequent cerebral opportunistic infections in HIV-positive patients, though their prevalence varies by geographic region. [1] While TBM and TE are common in HIV-infected patients, the simultaneous occurrence of both is extremely rare, even among those with advanced HIV.

TBM, caused by *Mycobacterium tuberculosis*, remains a leading cause of neurological morbidity and mortality in HIV-positive individuals. In regions with high tuberculosis (TB) endemicity, TBM accounts for up to one-third to one-half of all bacterial meningitis cases. [2] Individuals with concurrent HIV infection face a five-fold increased risk of CNS involvement and disseminated TB, with this risk being even higher in those with a CD4 count below 100 cells/µL [3].

Cerebral toxoplasmosis, caused by *Toxoplasma gondii*, is highly prevalent worldwide, with latent infection estimated to affect approximately one-third of the global population. [4] Among people living with HIV, millions are seropositive for *T. gondii*; however, only a subset develop active CNS disease, which manifests as brain inflammation and related neurological complications.

We introduce a case of TBM and TE in an HIV-positive patient with a CD4+ cell count of 27. Despite treatment with both antitubercular and antiparasitic therapies, the patient's condition continues to worsen, highlighting the complexity of managing dual CNS infections in patients with advanced HIV.

## Case presentation

A 46-year-old Caucasian female presented with a one-month history of constitutional fatigue, fever, and weight loss, along with confusion, headache, myalgia, lightheadedness, nausea, and vomiting. She denied seizures, photophobia, focal neurological deficits, visual disturbances, respiratory symptoms, recent TB exposure, or prior prophylaxis for opportunistic infections. She reported no significant dietary or animal exposure risks. Her medical history was notable for HIV-1 infection diagnosed 10 years prior, complicated by AIDS, with non-adherence to antiretroviral therapy (ART) over the past two years.

On presentation, her Glasgow Coma Scale (GCS) score was 15/15; she was alert and oriented to person and place but intermittently disoriented to time. Vital signs showed a temperature of 38.3°C, blood pressure of 115/70 mmHg, heart rate of 105 beats/min with regular rhythm, respiratory rate of 20 breaths/min, and oxygen saturation of 91% on room air. General examination revealed mild pallor and oral candidiasis without lymphadenopathy, hepatosplenomegaly, or skin lesions. Neurological examination demonstrated bilaterally reactive pupils of normal size, intact cranial nerves II-XII, and bilateral lower limb motor weakness. There were bilateral lower limb motor deficits, neck rigidity with flexion of the knees and hips (Brudzinski's sign), and bilateral Babinski's sign (upward extension of the big toes). Cognitive functions, including recent and remote memory, attention, and language, were preserved.

​​A lumbar puncture was performed for cerebrospinal fluid (CSF) analysis, with the results provided in Table 1. Nucleic acid amplification via GeneXpert *Mycobacterium tuberculosis*/rifampicin (MTB/RIF) (Cepheid, Sunnyvale, California, United States) detected TB without rifampicin resistance.

**Table 1:** Cerebrospinal fluid (CSF) analysis.

Component	Result	Reference range
Color	Clear	-
Transparency	Cloudy	-
Leukocytes in CSF	70 cells/mm^3^	0-8
Neutrophils in CSF	30	2% ± 5
Lymphocytes in CSF	65	62% ± 34
Monocytes in CSF	5	36% ± 20
Eosinophils in CSF	None	None
Glucose in CSF	0.89 mmol/L	2.8-3.9 mmol/L
Protein in CSF	1.23 g/L	0.18-0.45 g/L
Toxoplasma gondii IgG	800 IU/mL	Negative
Microbial examination	Acid-fast bacilli	Negative

**Figure 1:**
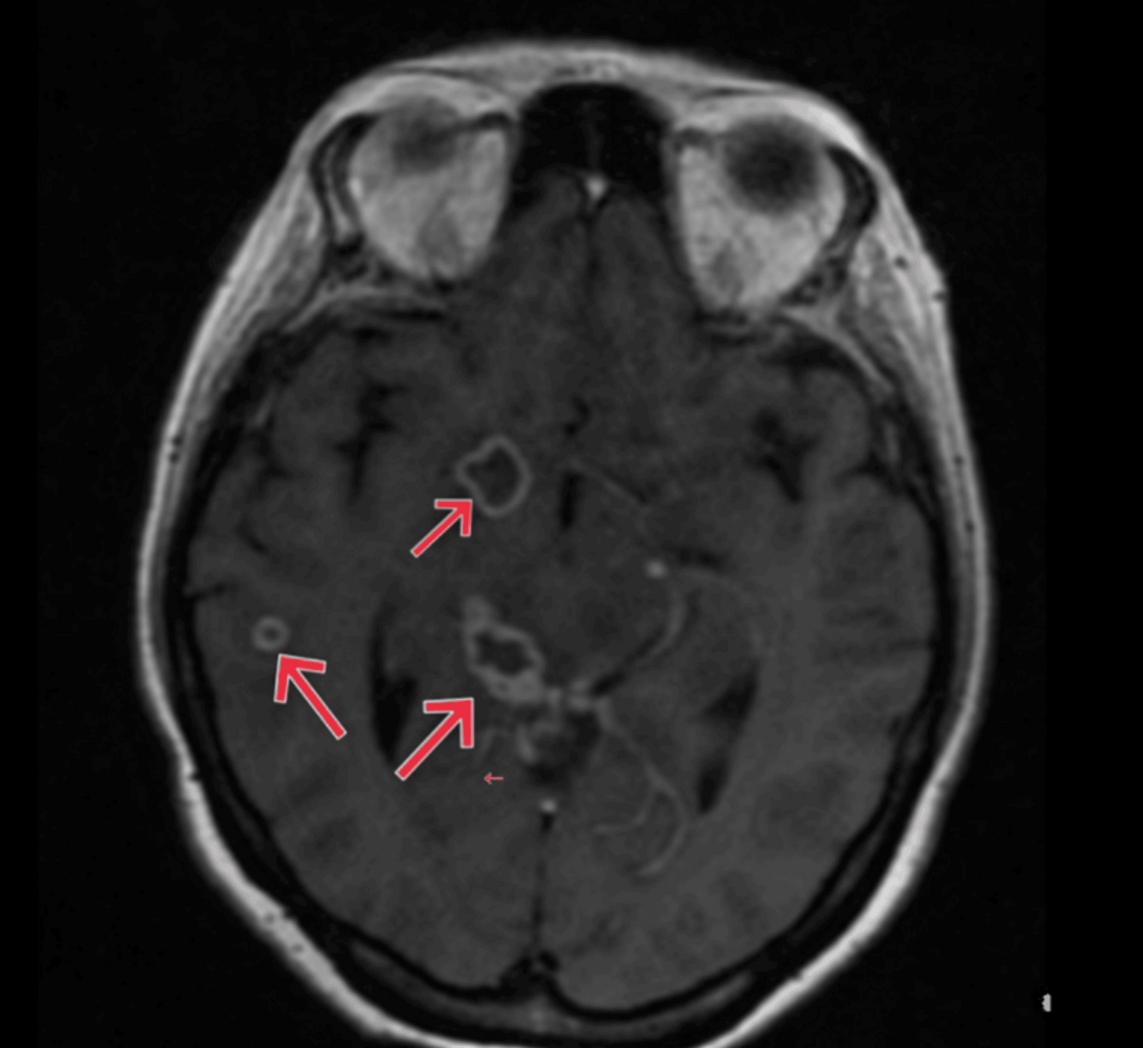
Axial post-contrast T1-weighted MRI showing multiple ring-enhancing lesions in the right cerebral hemisphere with surrounding vasogenic edema.

**Figure 2:**
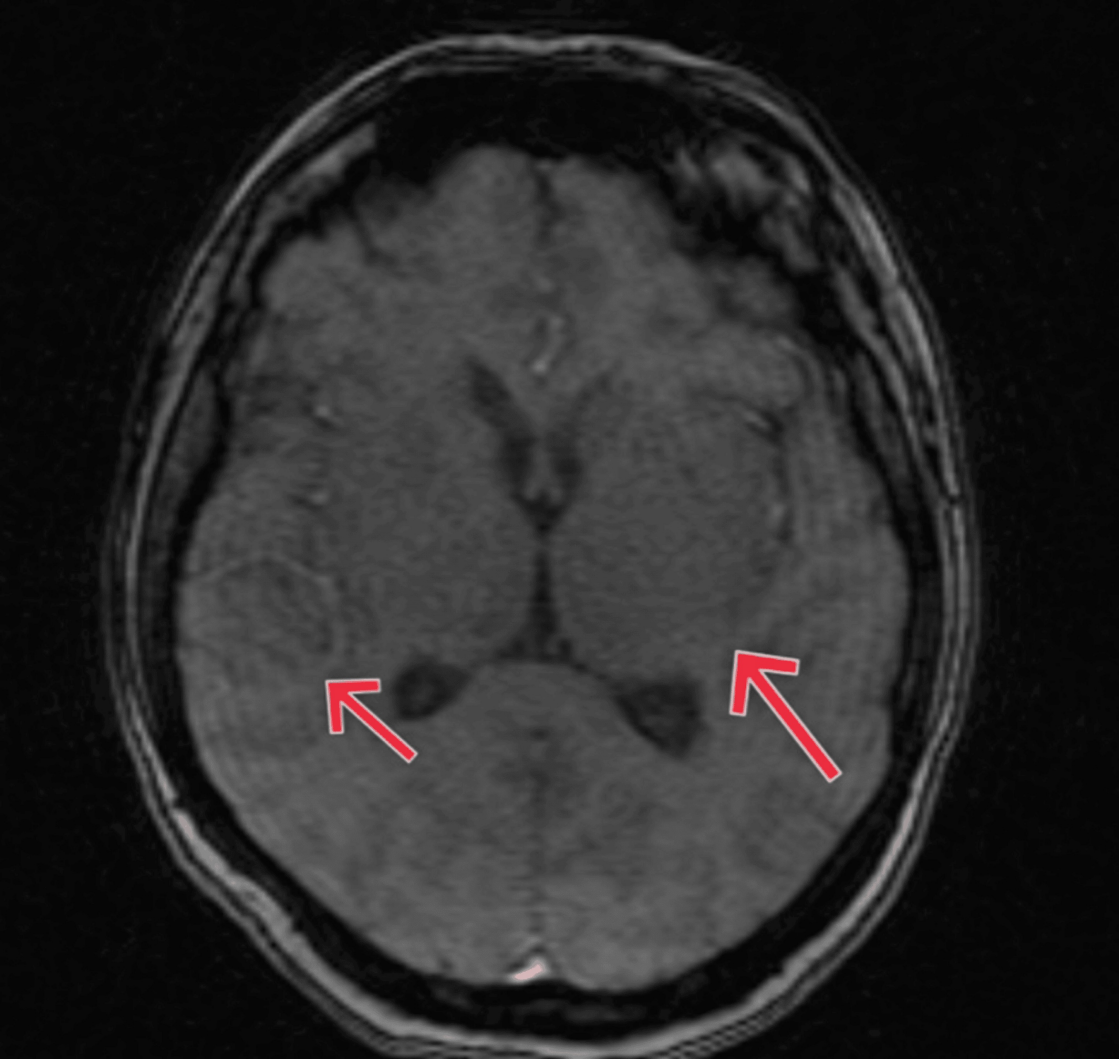
Axial fluid-attenuated inversion recovery (FLAIR) MRI showing bilateral hyperintense lesions in the basal ganglia (lentiform nuclei), consistent with edema.

A thorough microbiological evaluation of the CSF revealed *Mycobacterium tuberculosis* on GeneXpert testing and a markedly elevated IgG titer for *Toxoplasma gondii*. Additional CSF studies, including Gram stain, bacterial culture, India ink preparation, and cryptococcal antigen testing, were negative, helping to exclude bacterial meningitis and cryptococcal infection. Viral polymerase chain reaction (PCR) tests for herpes simplex virus (HSV) and cytomegalovirus (CMV) were also negative. Serum rapid plasma reagin (RPR) testing excluded neurosyphilis. Magnetic resonance imaging (MRI) of the brain showed multiple subcortical ring-enhancing lesions in the right temporal and left parietal lobes with associated vasogenic edema scattered throughout both cerebral hemispheres, including the bilateral parietal lobes, as shown in Figures 1, 2.

In advanced HIV, the principal imaging differential diagnosis for multiple CNS ring-enhancing lesions includes TE, tuberculous tuberculomas, and primary CNS lymphoma (PCNSL). TE typically presents with multiple small (<2 cm) ring-enhancing lesions, often in the basal ganglia and corticomedullary junction, frequently associated with marked perilesional edema. Tuberculomas, in contrast, may be solitary or multiple, often larger, with conglomerate ring enhancement and predilection for the basal cisterns and thalamus; they may demonstrate central T2 hypointensity due to caseation. PCNSL usually manifests as solitary or a few lesions, commonly larger than 2 cm, with homogeneous or irregular enhancement, periventricular or corpus callosal location, and may show diffusion restriction on diffusion-weighted imaging sequences.

Laboratory tests during the recent medical history showed low hemoglobin, thrombocytosis, high neutrophil count, elevated erythrocyte sedimentation rate (ESR), elevated C-reactive protein (CRP), low CD4 count, normal CD8 count, low CD4/CD8 ratio, and high viral load, as detailed in Table 2.

**Table 2:** Laboratory test results.

Test	Value	Normal value
Hemoglobin	105 g/L	120-140 g/L
Thrombocyte	462 × 10⁹/L	155-320× 10⁹/L
Neutrophil	14%	1-6%
Erythrocyte sedimentation rate (ESR)	40 mm/hr	1-15 mm/hr
Creatinine	78 µmol/L	60–110 µmol/L
C-reactive protein	5.89 mg/L	<0.6 mg/L
CD4+ T cell	27 cells/μL	404-1612 cells/μL
CD8+ T cell	806 cells/μL	220-1129 cells/μL
CD4/CD8 ratio	0.29	1-3.6
Viral load	26,600 copies/mL	Undetectable

Sputum acid-fast bacilli smear and culture were negative. No extrapulmonary foci of TB were detected on abdominal ultrasound or physical examination. Thus, there was no radiographic or clinical evidence of systemic TB outside the CNS at the time of presentation.

Considering the results from imaging and laboratory tests, treatment was initiated with a combined regimen. For TBM, the patient received rifampin 600 mg daily, isoniazid 300 mg daily, pyrazinamide 2000 mg daily, meropenem 2000 mg daily, linezolid 600 mg daily, moxifloxacin 400 mg daily, and adjunctive dexamethasone. Although GeneXpert testing did not reveal rifampicin resistance, linezolid and moxifloxacin were added empirically, given the severity of presentation and concern for potential multidrug resistance in the local epidemiological context. For cerebral toxoplasmosis, induction therapy consisted of trimethoprim-sulfamethoxazole 1440 mg three times daily for six weeks, followed by maintenance with pyrimethamine 50 mg daily, sulfadiazine 1 g every six hours (total 4 g/day in divided doses), and folinic acid (leucovorin) to prevent pyrimethamine-related bone marrow suppression. ART was deferred for six weeks after initiation of antitubercular therapy, consistent with current guidance for TBM to reduce the risk of severe central nervous system immune reconstitution inflammatory syndrome (CNS-IRIS). While toxoplasmosis alone would typically warrant earlier ART initiation within two weeks, the TBM recommendation generally prevails in cases of co-infection, requiring careful expert balancing.

Despite the continuous treatment for two months, the patient’s condition continued to deteriorate. Her CD4+ T-cell count has fallen to 26 cells/mm³. She experienced vomiting, a severe headache, and double vision, could not walk independently, and had a negative finger-to-nose test. Her speech was incoherent, and she was exhibiting signs of depression. New white lesions, a classic AIDS complication of oral thrush, were noted, and nystatin was prescribed for oral candidiasis.

The patient was discharged with ongoing treatment for TB, HIV, and maintenance therapy for toxoplasmosis. Upon discharge, her consciousness was minimally impaired, with occasional confusion, but she remained oriented to person and place, though not to time. Meningeal syndrome was mildly expressed, with the patient still experiencing some neck stiffness and intermittent headaches, particularly in the mornings. These symptoms, while less severe than during the acute phase of illness, persisted and caused discomfort, especially during periods of increased physical exertion or when in an upright position for prolonged periods. Motor deficits remained pronounced, particularly in the bilateral upper and lower extremities. The patient exhibited significant difficulty with ambulation, requiring a walker for support and assistance from a caregiver for mobility.

Long-term prognosis will depend heavily on her adherence to ART and the response to the ongoing treatment for TB and toxoplasmosis. While ART could help improve her immune function, her ability to mount an effective immune response may be compromised due to the significant degree of immunosuppression. Additionally, the complexity of managing dual CNS infections may further complicate recovery and rehabilitation efforts. Therefore, the patient will require a multidisciplinary approach involving neurology, infectious disease specialists, physical and occupational therapists, and psychological support to address ongoing medical needs, optimize quality of life, and provide palliative care if necessary. Objective follow-up should include serial neurologic examinations, hearing and vision screening, given the risk of sequelae post-meningitis, and repeat brain MRI and CSF analysis if clinically indicated. Monitoring adherence to ART and documenting functional outcomes using standardized measures such as the modified Rankin Scale (mRS) or Barthel Index (BI) are also recommended. In the event of clinical deterioration despite appropriate therapy, alternative explanations such as primary CNS lymphoma, immune reconstitution inflammatory syndrome (IRIS), or drug toxicity should be considered, and the role of brain biopsy discussed in the multidisciplinary team setting.

## Discussion

CNS opportunistic infections pose a substantial risk of morbidity and mortality. The likelihood of a specific etiology is influenced by factors such as CD4 count, age, ethnicity, risk behaviors, prior prophylaxis, and geographic region. Direct epidemiological data on the prevalence of simultaneous TBM and TE are extremely limited. In recent years, coinfection with *T. gondii* and TB has emerged as a growing public health concern in developing countries. Active TB infection reduces Th1 cytokine production while increasing Th2 cytokine release, resulting in suppression of cell-mediated immunity against *T. gondii* and thereby predisposing to reactivation or new infection [5]. In a cohort study conducted in Indonesia among HIV-positive individuals presenting with meningitis, 64 patients were evaluated: 21 (32.8%) were diagnosed with TBM, and an equal number (32.8%) had TE confirmed by CSF PCR. Notably, five patients (7.8%) demonstrated evidence of concurrent TBM and TE. While these findings provide an estimate of the frequency of dual CNS infection in this setting, they are derived from a single, geographically specific cohort and may not be broadly generalizable [6].

The unique clinical presentation and poor therapeutic response in this patient underscore the diagnostic and management challenges associated with such co-infections in the setting of profound immunosuppression. Clinical diagnosis of CNS infections in HIV-positive patients is inherently difficult due to overlapping symptomatology. TBM typically presents with subacute meningoencephalitic features such as headache, fever, altered mental status, and meningeal signs [7], whereas TE more often manifests with focal neurological deficits and seizures secondary to space-occupying lesions. In this case, non-specific symptoms such as fatigue, confusion, and headache were initially observed, but neuroimaging and CSF analysis were essential for establishing the dual diagnosis. MRI revealed multiple ring-enhancing lesions with perilesional edema, findings characteristic of TE but also consistent with tuberculomas. CSF analysis demonstrated lymphocytic pleocytosis and hypoglycorrhachia, features suggestive of TBM, and GeneXpert MTB/RIF confirmed the presence of *Mycobacterium tuberculosis*. Although *Toxoplasma gondii* IgG was elevated, serology alone cannot establish active CNS toxoplasmosis. In the context of advanced immunosuppression, the integration of imaging findings and clinical presentation supported the diagnosis of TE, though definitive confirmation generally requires clinical-radiologic correlation, therapeutic response, or PCR detection in CSF.

The patient’s severely reduced CD4+ T-cell count (27 cells/μL at presentation, later 26 cells/μL) and detectable viral load are consistent with advanced AIDS. CD4 counts below 100 cells/μL are strongly associated with increased risk for both TE and extrapulmonary TB, particularly TBM. At this level of immunosuppression, presentations are often atypical, disease progression is rapid, and responses to therapy are suboptimal. Despite receiving appropriate antimicrobial therapy for both TBM and TE, the patient experienced ongoing neurological decline, underscoring the critical role of host immune status in determining outcomes. In cases of deterioration despite appropriate therapy, alternative explanations such as primary CNS lymphoma, IRIS, or drug toxicity must be considered, and the role of brain biopsy discussed within a multidisciplinary team. Long-term prognosis will depend heavily on adherence to ART and the response to ongoing treatment for TB and toxoplasmosis. While ART can restore immune function, the profound degree of immunosuppression in this patient may limit recovery. Although early ART initiation reduces mortality in HIV-TB co-infection, starting ART in the setting of TBM or TE carries a risk of IRIS, which can exacerbate intracranial inflammation and worsen neurological outcomes [8]. For patients with TBM, immediate initiation of high-dose corticosteroids in conjunction with antitubercular therapy is recommended, while ART is generally deferred until clinical stabilization, neurological improvement, and normalization of CSF parameters are achieved, typically two to four weeks after starting TB therapy. This staged approach balances the benefits of immune recovery against the risks of CNS-IRIS [9]. In this case, the absence of ART for more than two years likely contributed to the progression of both infections; however, delayed reintroduction of ART due to concerns regarding IRIS may have further impaired immune recovery.

## Conclusions

This case serves as a vital reminder for clinicians to consider the possibility of multiple simultaneous opportunistic infections in patients with AIDS, particularly when there is an inadequate clinical response to standard treatment protocols. It also underscores the need for a broad diagnostic lens when interpreting CNS imaging and CSF findings in severely immunocompromised individuals. The simultaneous occurrence of TBM and TE, both of which are independently associated with high morbidity and mortality in HIV-positive patients, represents an exceptionally rare phenomenon. Ultimately, this case contributes to the growing awareness of complex CNS manifestations in advanced HIV infection and reinforces the importance of considering co-infections when clinical progression is atypical or unexpectedly severe. As the landscape of HIV care evolves, continued vigilance and case-based learning remain essential for improving patient outcomes in this vulnerable population.
